# From Simulation to Conversation: Simulated Patients’ Contribution to Meaningful Feedback Dialogues

**DOI:** 10.5334/pme.2247

**Published:** 2026-03-09

**Authors:** Annelies Lovink, Karlijn Geelkerken, Heleen Miedema, Jan-Joost Rethans, Walther van Mook, Marleen Groenier

**Affiliations:** 1 Department of Technical Medicine, University of Twente, Enschede, the Netherlands; 2 School of Health Professions Education, Maastricht University, the Netherlands; 3 Skillslab, Faculty of Health, Medicine and Life Sciences, Maastricht University, the Netherlands; 4 Department of Intensive Care Medicine, MUMC+, Maastricht, the Netherlands; 5 Academy for Postgraduate medical training, MUMC+, Maastricht, the Netherlands

## Abstract

**Introduction:**

Simulated patients (SPs) play a key role in medical communication training, yet their contribution to feedback dialogues during post-simulation feedback sessions remains underexplored. This study explored how SPs contribute to meaningful feedback dialogues among students during feedback sessions after simulation-based consultations.

**Methods:**

Using an interpretivist qualitative approach, video-recorded feedback sessions with first-year technical medical students and SPs were analyzed. Episodes identified as meaningful feedback dialogues were thematically analyzed, focusing on SP contributions.

**Results:**

Of the 120 one-minute episodes during feedback sessions, 36 episodes were classified as meaningful feedback dialogue. These were characterized by one or more of the following aspects: SPs’ guidance on task performance, shifting positions of SPs, and SPs supporting the contribution of students by facilitating and creating space.

**Discussion:**

SPs’ guidance on task performance appears to respond to students’ need for direction. While such guidance can support learning, it also risks reducing student reflection. Therefore, SPs need awareness of when and how to provide guidance. Although instructed to remain in the patient positions, SPs adopt multiple positions: the patient’s position, the expert’s position, and the meta-position during the feedback dialogue, which can enrich learning if managed consciously. Increasing SPs’ awareness of these different ways of contributing to students’ learning may enhance their educational impact in communication training.

## Background

Effective communication is a core component of medical education, often taught through consultations with simulated patients (SPs) [1]. SPs contribute significantly by providing feedback based on their unique perspective as patients, offering insights that complement teachers’ evaluations [2]. While the role of teachers in guiding feedback dialogues is well established, little is known about how SPs influence these feedback dialogues, particularly when teachers are not present, as is often the case during these discussions.

### Simulated Patients

Effective communication between patients and healthcare professionals is crucial for delivering high-quality care, as it contributes to better health outcomes and greater satisfaction for both patients and professionals [3]. Therefore, communication skills are considered core competencies within medical education programs. To foster effective learning environments, most medical schools incorporate SP programs into their curricula [4]. SPs contribute to an educational training and assessment setting in which students can improve their clinical and communication skills [1,3]. SPs play a crucial role by authentically portraying patient cases and by providing feedback from the patient perspective [1,5,6]. Although their backgrounds range from laypersons to trained actors, effective SPs are always trained to portray a patient consistently according to a role script, which contributes to a certain level of standardization during consultations across different learners [7]. SPs are known to contribute to meaningful student learning during the consultation itself [8,9].

Meaningful learning can be described as learning that is well-anchored and integrated into the learner’s cognitive structure, in contrast to rote learning, which is more reproduction-oriented [10]. In addition to portraying their role, SPs are trained to observe students’ behavior and to recall key aspects of the encounter accurately. This enables them to provide constructive feedback from the patient perspective during the feedback dialogues [11]. The value of SP feedback in supporting student learning has been well documented [2,12,13,14,15]. Although it is well established that SP feedback itself is valuable for students’ learning, much less is known about how SPs shape the feedback dialogue [8,16]. It remains unclear what SP’s specific contributions are to these feedback dialogues, despite these sessions being an important component of students’ learning [17,18]. Although the term simulated participant is also used in education and research, we choose to use the term simulated patients in this study, as all SPs portrayed patients, making the context immediately clear to readers.

### Feedback dialogues

SP-based communication training is frequently conducted in group settings, where students not only practice skills but also engage in group feedback dialogues afterward to reflect on their performance and share insights. Previous research has shown that teachers play a crucial role in supporting the quality of students’ group discussions through their discourse moves and interventions [19,20]. So-called discourse moves refer to the deliberate actions undertaken by a teacher intended to facilitate discussion [19]. Teachers’ discourse moves affect the discussion among students and, thereby, the quality of the dialogue [20]. While the role of teachers in guiding and moderating dialogues is well established, it remains unclear how the presence and behavior of SPs affect the quality of these feedback dialogues. Understanding the latter is crucial, as SPs constitute another vital, yet poorly explored part of the students’ learning process. While the specific influence of SPs on feedback dialogues remains unclear, previous research has identified key general factors that contribute to meaningful group discussions in educational settings. To positively influence the student’s learning outcomes, group discussions may benefit from including high-transactive dialogues [17,18].

Dialogues are referred to as high transactive when the students engage with and build upon each other’s ideas [17,21]. High-transactive dialogues are fostered when participants actively engage in discussions by elaborating on each other’s viewpoints, integrating new insights and co-constructing knowledge [22,23]. These interactions involve critical thinking, synthesis and dynamic exchange of perspectives [17,24]. In contrast, dialogues with low-transactive episodes lack depth in the interaction, meaning they consist mainly of isolated statements or surface-level agreement [22,23].

In this study, we conceptualize meaningful feedback dialogues (MFD) during student group discussions as interactions containing high-transactive moments, characterized by reflection and mutual building of ideas, where students demonstrate deep engagement and learning through interactive discourse [10,25,26].

Building on these insights into what fosters meaningful group discussions, positioning theory offers a useful framework to explore how positioning of SPs within these interactions may influence the dynamics and outcomes of group discussions. Positioning theory posits that individuals both assume and assign roles to themselves and others within specific institutional or interpersonal contexts [27]. Using positioning theory, Sargeant et al. (2017) have shown that SPs can take on different positions during the consultation which shape the interaction with the student, including positions as patients, trainers or even parental figures [28]. We expect that these different SPs positions may be carried over, consciously or unconsciously, into the feedback dialogues.

In summary, although previous studies have recognized the role of SP feedback in student group discussions, the specific influence of SPs on these feedback dialogues has not yet been examined through the theoretical frameworks of transactivity and positioning theory. Moreover, the focus of this study is on settings without a teacher, allowing the interaction between the students and the SP to be examined in isolation. Understanding how SPs contribute to these feedback dialogues promises to provide valuable insights for optimizing SP-based communication training.

Consequently, this study aims to answer the question: “How do simulated patients contribute to meaningful feedback dialogues (MFD) among students during feedback sessions after simulation-based consultations?”

## Methods

### Study Design

A qualitative approach was adopted [29], using an interpretivist research paradigm to analyze video recordings of feedback dialogues between students and simulated patients following SP-based consultations. This approach enabled an in-depth exploration of how SPs may have influenced group dialogue after the consultation. Thematic analysis was used to analyze the data, allowing us to identify patterns and variations in interaction within these feedback dialogues [30].

### Context and setting

This study was conducted during communication education sessions for first-year Technical Medicine students at the University of Twente (the Netherlands), in which a student engages in a conversation with an SP, while peers are present as observers. These students will become technical physicians, licensed healthcare professionals who contribute to optimizing patient care using technology [31]. Their role centers on integrating technological innovations into direct patient care, and they are legally licensed to independently conduct medical consultations and perform certain procedures [31]. As technical physicians are legally authorized to perform some medical interventions, communication skills training is an integral part of the three-year bachelor program. Throughout the bachelor’s curriculum, on average students participate in 22 SP-encounters to practice communication skills ranging from basic consultations to complex conversations, such as breaking bad news.

In these sessions students engage in SP-encounters without a teacher present, and on the same day also participate in at least one encounter in which a teacher joins the feedback dialogue. In both situations, the post-encounter conversation is structured as a feedback dialogue centered on reflection, involving both the student and peers. These dialogues follow a fixed sequence: first, the student reflects on their own performance, second, the SP provides feedback from the patient’s perspective and third, peers contribute their observations and when present, an educator adds further reflection.

In this study, we deliberately described the activity as a feedback dialogue rather than debriefing, a term commonly uses in team simulation training. Although the differences are subtle, the nuances lie primarily in the structure of the conversation and the presence of a facilitator. Debriefing typically involves a facilitator who guides the session according to a structured reflective model, aimed at learning from a simulation in general as a team [32]. In our setting, such a facilitator is not always present. While participants are familiar with the general structure of the feedback conversation, the absence of a facilitator allows for a more flexible, student-led, individually tailored conversational approach, focusing on the development of the individual student.

The 40 SPs at the University of Twente are laypersons whose experience as SPs ranges from 1 to 20 years. They work on a non-contract basis and are between 30 and 70 years of age, with a male–female distribution of 30/70. SPs participate biannually in role-play and feedback skills training sessions, supplemented by four annual role-specific sessions on assessment scenarios. Training emphasizes acting techniques such as emotional expression, consistency, and credibility. In addition, SPs receive feedback training in which SPs learn to provide patient-perspective feedback grounded in their perception and experiences of the students’ behavior. Importantly, SPs are not trained as educators. Their training deliberately and explicitly emphasizes maintaining the patient’s perspective in feedback, thereby supporting student reflection while avoiding a teaching or instructive role.

**Figure 1:**
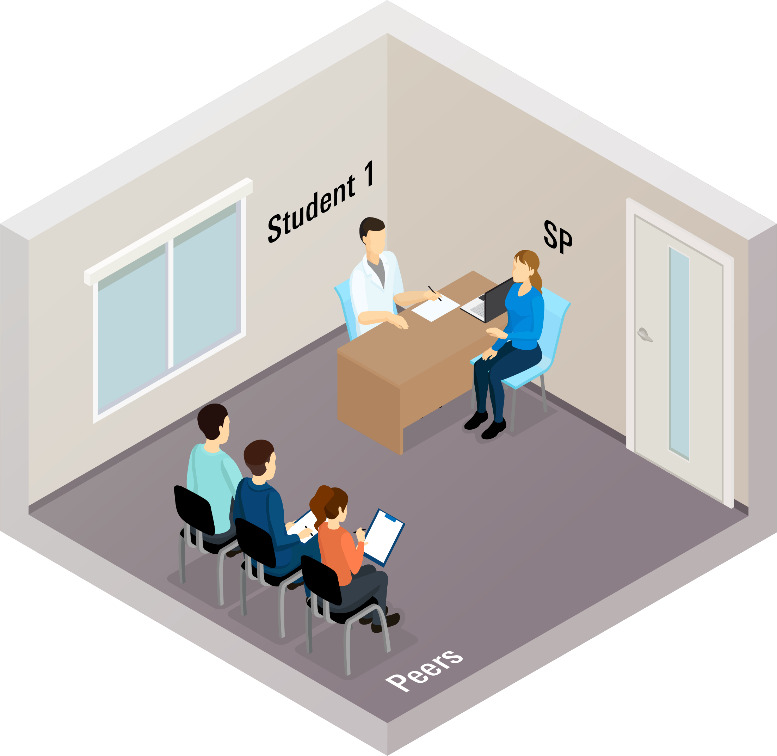
Educational setting with student, SP and peers.

All encounters are video recorded using Learning Space [33], capturing not only the consultation itself but also the feedback dialogue between the students and SP. Figure 1 represents the setting for both the consultation and the feedback dialogue.

### Data collection

The raw dataset consisted of 597 video recordings of SP-student encounters varying in duration from three seconds to three hours, originally collected for educational purposes. The videos were recorded between September 8, 2022, and June 1, 2023, and involved first-year students (n = 126). After correcting for recording errors (not correctly starting or ending recording at the appropriate time), most videos had a duration range of 5 to 10 minutes. To minimize any potential influence on the dialogue and to preserve its authenticity, written informed consent from both students and SPs was obtained retrospectively.

### Data selection

**Figure 2:**
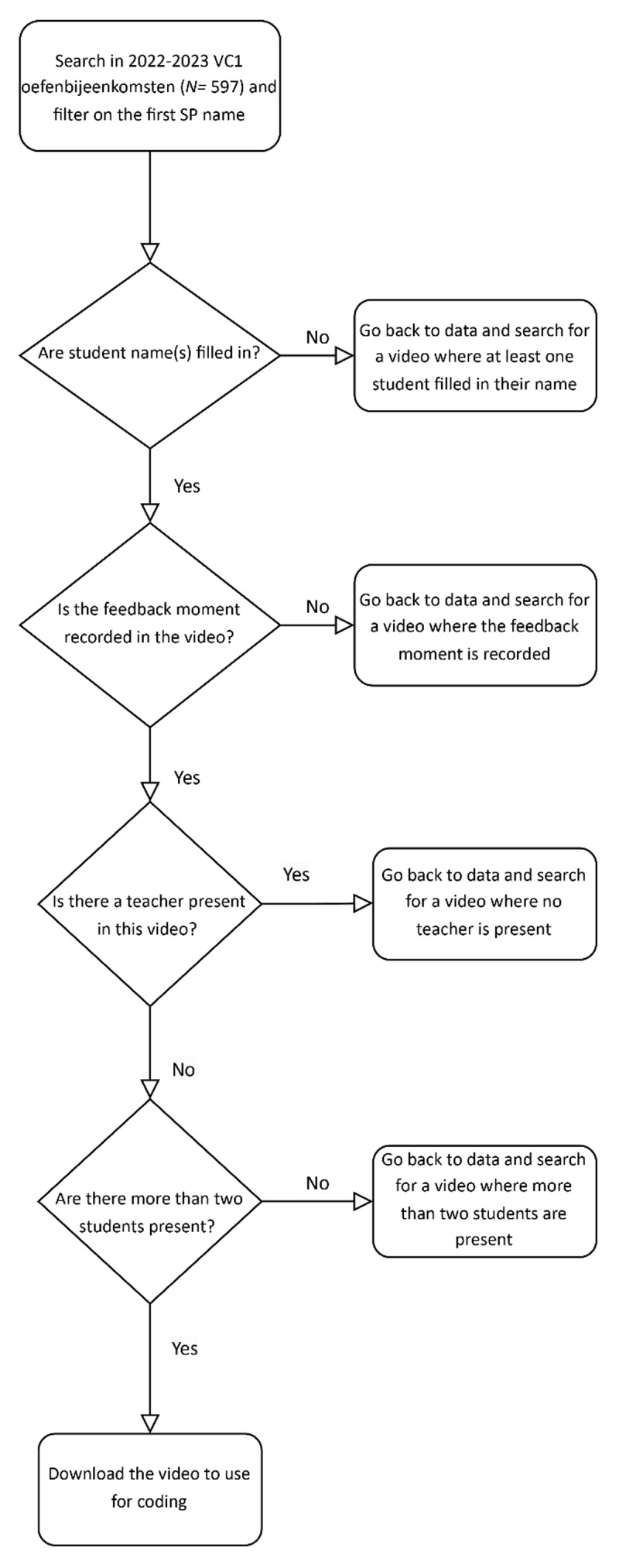
Overview of the data selection process.

As the aim was to explore how SPs in general contribute to feedback dialogues, variation in SPs was essential in this study. Therefore, a broad range of unique SPs were included, and an adapted stratified sampling approach was employed to ensure a maximally diverse representation of SPs. In addition, a purposeful sampling strategy was used to select recordings relevant to this study. Recordings were included if they captured the complete feedback moment, including the group discussion. Although the consultation itself was not central to this study, it was nevertheless recorded in all cases. Furthermore, inclusion required recordings to feature at least three students: the student who had conducted the consultation and a minimum of two peers, as this reflects the typical group setting in the communication training sessions. Recordings were excluded if the names of the students or SP were missing, or if the feedback moment with group discussion had not been recorded. Additionally, recordings in which a teacher was present were excluded, as a teacher could potentially influence the dialogue, introducing a variable that would interfere with answering the research question. This was the case in approximately half of the available recordings. Figure 2 provides an overview of the data selection process.

### Data processing

Videos were systematically segmented into one-minute episodes. A pilot with three videos confirmed that one-minute episodes contained sufficient instances of high-transactive episodes to enable analysis. One-minute episodes helped preserve the richness of the interaction and allowed for a more detailed analysis [22]. Shorter segments did not allow enough room for turn taking, while longer segments complicated analysis, due to the complexity and overlap of multiple conversational moves, which reflect the frequency of turn changes. Next, the episodes were analyzed by two researchers (KG and AL) to determine whether the episode was predominantly ontask. An episode was classified as ontask if more than 30 seconds of the one-minute segment focused on the consultation, and as offtask if 30 seconds or more concerned other topics. The two researchers independently assessed all episodes and subsequently compared their judgments to reach consensus.

**Figure 3:**
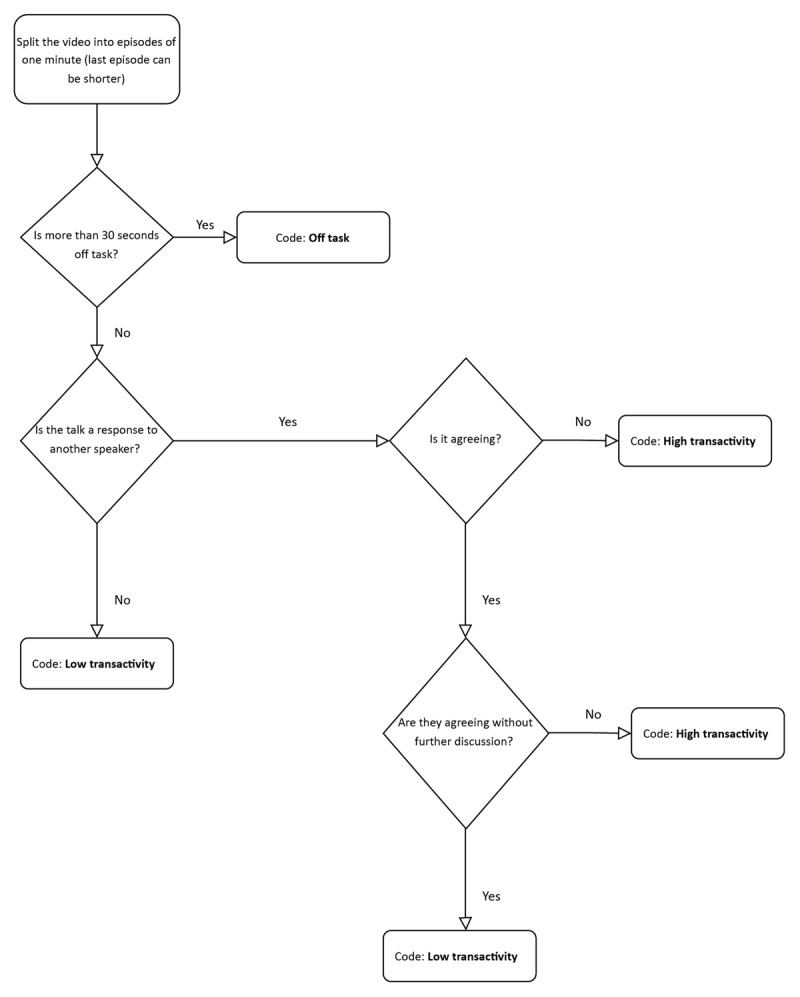
Overview of the data processing process.

When the episode was ontask, it was further categorized into high transactive or low transactive, also coded by KG and AL. The coding process for high-transactive episodes (and thus for MFD) followed a structured hierarchical approach. The analysis began by determining whether a student’s statement was a response to another speaker, which could be either an SP or another student. Statements without responses were classified as low transactive. Episodes with responses were coded as high transactive when discussion occurred and as low transactive when no discussion took place. The researchers independently coded the episodes and then discussed their analyses until consensus was reached. Figure 3 illustrates the steps involved in data processing process.

### Analysis: thematic analysis

Subsequently we conducted a thematic analysis of the one-minute episodes identified as meaningful, i.e., high-transactive episodes, feedback dialogues [30,34]. This qualitative step allowed us to examine the substantive content of the interaction [35]. First, the one-minute episodes identified as high transactive and therefore as MFD were independently coded by the same two researchers (KG and AL), applying principles of open coding, using Atlas.ti. [29]. This coding process involved identifying key elements of interaction in individual episodes by repeated reading and constant comparison. Data were reviewed independently, followed by a collaborative discussion among the two researchers about the appropriateness of the codes. Next, patterns within the coded data were examined to identify recurring themes. Constant comparison was used across different episodes to refine identified themes. Finally, overarching themes were established with the involvement of a third researcher (MG), who assisted in synthesizing the findings and resolving discrepancies. This iterative constant comparison approach ensured reliability in capturing the characteristics of MFD.

### Reflexivity

The researchers acknowledged their position and the potential bias that comes with it. Reflexivity was consequently applied throughout the study to make these positions and the associated perspectives explicit and to critically consider how they influenced data analysis, in line with an interpretivist paradigm. Three researchers in this study hold an insider position regarding the subject. Researcher AL is a lecturer and SP educator at the department of Technical Medicine (TM) with 15 years of experience working with SPs. MG is a lecturer and researcher at the department of TM with substantial experience in human and non-human simulation education. Researcher KG is a lecturer and one of the teachers of the student group used for this study. While aware that their insider positions may shape interpretations, the researchers also recognize the advantages of having an a priori, more in-depth understanding of communication training with SPs. In addition to the three insiders, two outside researchers were involved in the study. JJR is a professor in the field of human simulation and has worked with SPs since 1985. WvM is a clinician and a professor in the field of medical education with extensive experience in (qualitative) research and supervising PhD candidates in various aspects of medical education and medicine. Because assumptions and expectations of the three researchers that hold an insider position may have unconsciously influenced data analysis and interpretation, these three researchers explicitly applied reflexivity. After each phase of analysis, they discussed their progress and interpretations every two weeks. Additionally, JJR and WvM were involved in the final stage of the thematic analysis to assess the coherence of the themes and quotes. Their perspectives contributed to a careful and shared interpretation of the findings.

### Ethical approval

This study was ethically approved by the BMS Ethics Committee of the University of Twente (number 230861).

## Results

**Figure 4:**
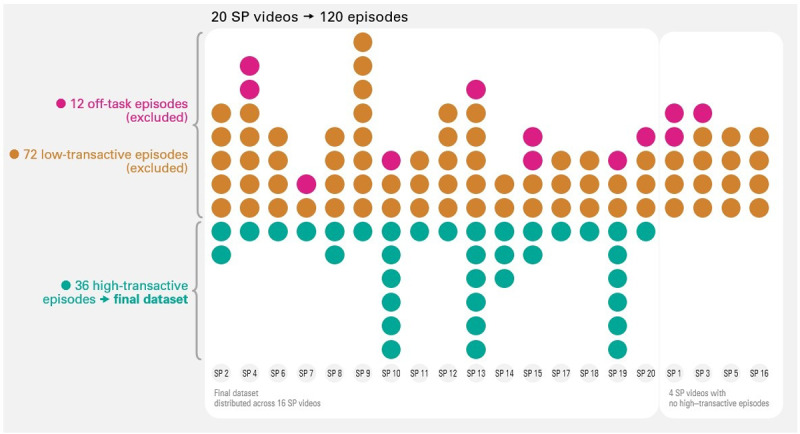
Distribution of episodes across feedback dialogues per SP.

After applying the inclusion and exclusion criteria, 20 videos were selected for analysis, each featuring a different SP, as no additional unique SPs were available. Overlapping SPs were avoided to prevent confounding effects. The segmentation of the 20 videos resulted in a total of 120 episodes of which 36 episodes (33,33%) were classified as high-transactive episodes, referring to MFD in which students built on each other’s reasoning. These episodes occurred in 16 (80%) of the 20 feedback dialogues with SPs (Figure 4). The green row of Figure 4 represents the final dataset used for further analyses, excluding off-task and low-transactive episodes. The distribution of high-transactive dialogues among SPs shows considerable variation. This variation likely reflects contextual factors beyond the SPs’ influence, such as student group composition, consultation timing, SP case, training phase, or teacher presence in prior sessions.

Through thematic analysis of the episodes classified as MFD, characterized by high-transactive episodes, three themes were identified that provided insight into the content of the interactions. MFD were characterized by one or more of the following aspects: SPs’ guidance on task performance, shifting positions of SPs, and SPs supporting the contribution of students by facilitating and creating space. The following section describes the three themes separately and provides context by presenting quotes from different episodes. Each episode is labeled with a number referring to the SP and (a part of) the high-transactive episode (e.g., 10.5 refers to high-transactive episode 5 of SP 10). When multiple peers were involved in a quotation, they were numbered to ensure clarity.

### SPs’ guidance on task performance

The first theme centers on the feedback provided by the SPs, in which they guide students on how tasks should be performed correctly and highlight actions that meet or fail to meet expectations. These inquiries arise from both the SPs, who provide guidance on how tasks should be performed, and the students, who seek guidance for the correct approach. This theme reflects a dynamic interaction in the feedback process. The following episode illustrates how the SP provided direct guidance on the task, while students actively engage with and acknowledge this guidance:

*SP 10: You want to… and I have often noticed with students that they indeed want to maintain a certain level of politeness, saying things like “hmm” and “okay” in between. But you just kept looking at me, and I thought, “Oh, just a little longer, and she’ll be in a sort of…”. You really should have intervened earlier*.

*Student: Yes*.

*SP 10: Yes, I think so*.

*Student: Yes*.

*SP 10: You should have intervened earlier in what you were saying about, um…*

*Peer 1: Yes, those were good sentences*.

*Peer 2: You can also just state it directly, like, “I’d like to learn a bit more about your complaint so we can help you properly. So I’d like to ask a few more questions.” That way, it might also be clearer for the patient that this is what we are here for. And the nice stories about my son are fine too, but that’s not the main focus*.

*SP: I did enjoy it, but I am also a fairly kind person who likes to chat a lot*.

*Peer 2: Yes, I understand*.

*SP 10: If you interrupt me too quickly, that’s not good either, because then I don’t feel heard*.

*(Episode 10.5)*

In addition to guiding the student on how to perform tasks correctly, the SP also provided guidance on what should be avoided. In the episode below, the SP points out how the student downplays the symptom using minimizing language:

*SP 11: Just for a bit, you don’t say ‘just for a bit’*.

*Student: Did I say that?*

*Peer 1: Yes*.

*Student: Oh*.

*SP 11: You need to be careful with those kinds of things, making sure you don’t say them*.

*Student: Yes, that’s true. No, I shouldn’t say ‘just a bit’. Oh, I didn’t even realize I said that*.

*SP 11: That’s what I mean, you probably didn’t notice it yourself, but you need to be mindful of those little things*.

*Student: Okay, I’ll keep that in mind for next time*.

*Peer 1: Other than that, you had a clear understanding of what he was saying. He was summarizing again at the end as well. Yes*.

*Student: Okay, great*.

*(Episode 11.1)*

### Shifting positions of SPs

Another theme identified during MFD is shifting positions of the SPs. These included the patient position, where SPs provided feedback from the patient’s point of view based on their experience during the consultation; the expert position, where SPs offered content-related guidance focusing on medical and communication skills; and the meta-position, where SPs reflected on their role in the consultation or offered insight into the broader curriculum. These positions were not fixed but could alternate throughout the meaningful dialogue. For clarity, we provide examples of each position separately.

**Patient position**

The SP adopted the position of a patient and offered feedback based on how they experienced the consultation as a patient. In the following episode, the SP commented positively on how the student created space for the patient to speak and communicated clearly about next steps:

*SP 17: Somehow, you also got me to talk; you gave me space to speak as well*.

*Student: Yes*.

*SP 17: So that was really nice. Uhmm, at the end, you clearly said, “I will discuss it with the GP,” and it was very clear, something like in a week and a half or a week, I don’t remember exactly what you said. But then you made sure that contact with the GP would happen and that you would follow up on it. So, I know that something is going to happen, which I really appreciate. And you are an intern, I can’t imagine that an intern would say, “Well, I’ll prescribe you a few tablets.” So, in that sense, I found it a very pleasant conversation*.

*Student: Okay, yes*.

*(Episode 17.1)*

**Expert position**

In this position, the SP provided guidance that extended beyond the patient position and included content-related topics. The SP adopted an instructive stance, drawing on domain-specific knowledge and familiarity with the consultation format to offer advice. The following episode illustrates how the SP offered concrete suggestions for managing the flow of conversation, adopting an expert position. Toward the end of the episode, the SP briefly shifted to a patient position, highlighting how multiple positions could coexist within a single episode:

*Student: Yes, I get that. I was listening very attentively and trying to take it all in*.

*SP 10: Yes, but I was actually saying way too much, right?*

*Student: Yes*.

*SP 10: So I think it would be good to practice interrupting, maybe repeating something I said or using my breathing pauses, because I do have to breathe sometimes, to bring the conversation back to your own plan. I think that’s something you could work on. But I really liked that I got to talk so much. And it’s not like I don’t know why I’m at the doctors. I do know that*.

*(Episode 10.2)*

**Meta-position**

In this position, the SP reflected on how their role was enacted during the consultation and provided guidance on the student’s responsiveness to general cues within the interaction. The following episode demonstrates how the SP comments on where in the consultation the student could have been more curious and attentive to subtle hints:

*SP 20: Yes, yes, and here and there, you could be a bit more curious, right? Like when I mentioned that my wife reacted to it, like “this is where it comes from.” Or when I hinted at smoking, and I don’t think you picked up on that. No*.

*Student: No, I think I missed that*.

*Peer: I wanted to ask that*.

*Student: Yes, I missed that*.

*SP 20: Yes, I said something like, “Well, my wife is angry because, yes, this is what happens.”*

*(Episode 20.1)*

Beyond reflection on their role in consultation sessions, SPs also reflected on the role of SP consultation sessions in the students’ broader educational context. The following episode illustrates how the SP comments on what students can expect in later stages of their education:

*SP 13: Yes, that’s true, and this consultation is more about the contact, and the other things are just much more… you’ll get more into that later*.

*(Episode 13.2)*

### Facilitation and creating space

In 10 episodes, SPs took a more passive role, simply facilitating the discussion or creating space without actively intervening, allowing room for reflection. The following episode exemplifies how an SP facilitated a conversation by prompting students to share their thoughts and encouraging peer feedback:

*SP 6: Well, what did you think of it yourself?*

*Student: Uh, yes, I found it difficult because with diabetes, you have symptoms, but you can’t really ask about things like pain, where you could still ask where exactly it is. But with diabetes—or at least, I read that they thought it was diabetes—uh, yes, I find it difficult to really go deep into the complaint. Yes, and other than that, I think it went fine, but sometimes I struggled to find the right words*.

*SP 6: Hmm Hmm (SP agrees with the student)*

*Student: Yes, that a little bit*.

*SP 6: Well, let’s hear what the audience thinks*.

*Peer 1: Uh, yes, overall, I thought it was good, uh, just a few small things. For example, when you went to pick him up, you said, uh, Mr. Duurstra… I think it could be a bit more confident, but…*

*Student 6: Yes, yes, but he was in a conversation… so I thought maybe it was something else, so…*

*Peer 1: Yes, apart from that, I thought it was a good introduction. You did include the elements you were given last time*.

*(Episode 6.1)*

When the SP remains silent and refrains from intervening, space emerges for students to engage directly with one another’s reflections. This stimulates an active dialogue, in which students explore, question, and negotiate different perspectives without the SP’s direct involvement.

## Discussion

This study aimed to explore the contribution of SPs to MFD during feedback sessions after simulation-based consultations. This study enhanced our understanding of SPs’ contribution to MFD. The findings indicate three key aspects of MFD: SPs’ guidance on task performance, their ability to shift positions, and the creation of space for reflection, which will be discussed in more detail below.

### Students’ need for guidance

Previous research has shown that SPs’ feedback is highly valued and can support the development of communication skills [2,11,36,37]. One key finding of the current study is that SPs often adopt a guiding role in feedback conversations, offering explicit advice on what students should do or should avoid. This is notable given that, in the context of this study, SPs were specifically trained to provide feedback from their patient perspective, focusing on how the interaction felt to them as the patient [16]. To address potential ambiguity in terminology, we clarify that in our context feedback refers to the articulation of the SP’s lived experience during the encounter from the patient’s perspective, whereas guidance refers to suggestions or advice aimed at improving future performance. Although SPs are trained in giving feedback from the patients’ perspective, they often tend to give advice. This tendency to offer guidance may be explained by the fact that many SPs in this study were highly experienced and had developed a clear sense of what effective communication entails. Over time, this experiential knowledge may result in more instructive or normative forms of feedback.

The shift toward more instructive or normative contributions by SPs suggests that SPs may also respond to students’ implicit or explicit requests for guidance, particularly when students are still seeking clarity on what constitutes “correct” performance. First-year students are still developing core communication skills and rely heavily on external guidance. Their focus on task performance may invite more direct forms of feedback. While students may seek clarity, it is questionable to what extent SPs are individually equipped to provide accurate, consistent content-based advice. Moreover, encouraging students to engage in peer discussion about what constitutes effective communication may be more conducive to learning than receiving definitive answers [38]. While guidance can support learning, it also risks reducing student reflection. These insights indicate that it may be valuable for SP training to encourage reflection on how prescriptive instruction and normative guidance is applied and what effects it may have.

### Shifting positions during and after the consultation

Previous research has shown that SPs may shift positions during the consultation [8,28]. The results of the current study expand this understanding by demonstrating that such shifts also occur during the feedback session. Specifically, three distinct positions were identified: the patient position, the expert position, and the meta-position. This fluid movement between positions during feedback dialogues aligns with findings from Lovink et al. (2024) showing that SPs contribute to learning by adopting different positions during the consultation [8]. Building on this study, where shifts in SP positions were described during the consultation, the current study shows that during feedback sessions, students appeared to benefit from the SPs’ varying contributions from different positions, as such moments were identified as MFD.

At the same time, this flexibility highlights the importance of SP awareness. Without a clear understanding of the distinct nature and purpose of each position, there is a risk that the patient position, the core of the SP’s unique contribution [2], may become diluted. Feedback rooted in the patient position offers insights that neither faculty nor peers can provide. Therefore, in SP training it can be useful to foster awareness of these different roles and explicitly support SPs in maintaining the patient position as a central and consistent anchor within group dialogue. Helping SPs develop awareness of the different positions they may adopt, while maintaining their patients’ positions, can enhance the educational value of feedback dialogues.

### The value of creating space for reflection

The third theme highlights the value of a facilitative or even passive stance taken by SPs in MFD. By (intentionally) stepping back, SPs can create space for students to engage in peer discussion, take ownership of their learning, and reflect. This more restrained presence can be just as impactful as feedback, enabling students to construct meaning through submersion and interaction rather than instruction.

These findings underscore that during MFD it is not solely about the content of feedback, but also about how SPs use their presence, consciously or unconsciously to support the learning process. Sullivan et al. (2023) described how SPs perceive themselves as active contributors to student learning, making deliberate choices in how they engage based on what they feel the situation demands [37]. Our study shows that this responsive stance extends into the feedback phase: SPs do not simply “give feedback” but adapt their level and nature of involvement based on group dynamics and student’s needs. Therefore, it can be important to make SPs aware of the students’ level, year, and specific learning objectives for each session.

Prior research on small group learning shows that teachers foster productive discussions through subtle strategies such as backchanneling and creating space for peer exchange [20,39]. Similarly, SPs in our study facilitated reflection not only through their words but through how they positioned themselves, sometimes by intervening, other times by stepping back. This facilitative presence of the SP, whether active or restrained, appears to be a key finding during MFD. These insights support the importance of preparing both SPs and students for this kind of education intervention.

### Challenges and strengths

A strength of this study lies in the fine-grained analysis of naturally occurring interactions, which showed how SPs contribute to MFD and support learning. However, several challenges should be noted.

The occurrence of MFDs was not evenly distributed across the SPs. Several factors may account for this variation. One possible explanation concerns individual differences among SPs. SPs with greater experience or a more verbally expressive communication style may be more inclined to actively guide students. Differences among student groups are also likely to contribute. Some groups may display higher levels of interactivity and openness to peer reflection, whereas others may engage in feedback in a more reserved or task-oriented manner. In addition to these interpersonal factors, contextual elements may also have influenced the emergence of MFDs. The timing of the feedback session, for instance, could play a role; sessions scheduled later in the day may be characterized by reduced energy and attention among students. Similarly, limited time for discussion may constrain opportunities for elaboration. Furthermore, the presence of a teacher in a previous session might have affected students’ need for discussion.

Another consideration is that framing the activity as a feedback dialogue allowed for flexible, student-led reflection, while still sharing elements of debriefing. However, the absence of a facilitator may have affected the depth of reflection, and it remains unclear how facilitator involvement would influence learning.

The study was conducted with first-year students, whose need for clarity may have influenced the directive nature of SP feedback. It would therefore be valuable to replicate this study with more advanced students to explore whether experience and training impact their need to seek clarity.

Moreover, the familiarity of SPs with the educational context may have shaped their behavior, which could affect how transferable these findings are to other settings. We promote transferability by describing the findings and the context in detail and discussing our findings in the light of existing literature from different settings. Future research with more diverse learner groups and settings could help determine how these dynamics evolve with increasing student experience and varying SP training approaches.

## Conclusion

In conclusion, SPs might contribute to meaningful feedback dialogues in three main ways: by providing guidance, by shifting between different positions, and by creating space for students’ reflection. Increasing SPs’ awareness of these different modes of contribution offers the potential to enhance their educational impact in communication training. This underscores the importance of targeted SPs’ training that helps them balance guidance with space-giving, manage their multiple and different positions effectively, and align their contributions with students’ learning needs.

## Data Accessibility Statement

The datasets used and analyzed during the current study are available from the corresponding author on reasonable requestt, due to privacy considerations and the small, identifiable participant group.

## Figures and Tables

**Table:** 

SP	Simulated Patient
TM	Technical Medicine
MFD	Meaningful Feedback Dialogue
